# Macrophage Activation and Differentiation Signals Regulate Schlafen-4 Gene Expression: Evidence for Schlafen-4 as a Modulator of Myelopoiesis

**DOI:** 10.1371/journal.pone.0015723

**Published:** 2011-01-07

**Authors:** Wendy J. van Zuylen, Valerie Garceau, Adi Idris, Kate Schroder, Katharine M. Irvine, Jane E. Lattin, Dmitry A. Ovchinnikov, Andrew C. Perkins, Andrew D. Cook, John A. Hamilton, Paul J. Hertzog, Katryn J. Stacey, Stuart Kellie, David A. Hume, Matthew J. Sweet

**Affiliations:** 1 The University of Queensland, Institute for Molecular Bioscience, Queensland, Australia; 2 The Roslin Institute and Royal (Dick) School of Veterinary Studies, University of Edinburgh, Roslin, United Kingdom; 3 Department of Medicine, The University of Melbourne, Royal Melbourne Hospital, Parkville, Australia; 4 Centre for Innate Immunity and Infectious Diseases, Monash Institute of Medical Research, Monash University, Clayton, Australia; 5 The University of Queensland, School of Chemistry and Molecular Biosciences, Queensland, Australia; 6 The University of Queensland, Australian Infectious Diseases Research Centre, Queensland, Australia; Singapore Immunology Network, A*STAR, Singapore

## Abstract

**Background:**

The ten mouse and six human members of the *Schlafen* (*Slfn*) gene family all contain an AAA domain. Little is known of their function, but previous studies suggest roles in immune cell development. In this report, we assessed *Slfn* regulation and function in macrophages, which are key cellular regulators of innate immunity.

**Methodology/Principal Findings:**

Multiple members of the *Slfn* family were up-regulated in mouse bone marrow-derived macrophages (BMM) by the Toll-like Receptor (TLR)4 agonist lipopolysaccharide (LPS), the TLR3 agonist Poly(I∶C), and in disease-affected joints in the collagen-induced model of rheumatoid arthritis. Of these, the most inducible was *Slfn4*. TLR agonists that signal exclusively through the MyD88 adaptor protein had more modest effects on *Slfn4* mRNA levels, thus implicating MyD88-independent signalling and autocrine interferon (IFN)-β in inducible expression. This was supported by the substantial reduction in basal and LPS-induced *Slfn4* mRNA expression in IFNAR-1^−/−^ BMM. LPS causes growth arrest in macrophages, and other *Slfn* family genes have been implicated in growth control. *Slfn4* mRNA levels were repressed during macrophage colony-stimulating factor (CSF-1)-mediated differentiation of bone marrow progenitors into BMM. To determine the role of *Slfn4 in vivo*, we over-expressed the gene specifically in macrophages in mice using a *csf1r* promoter-driven binary expression system. Transgenic over-expression of *Slfn4* in myeloid cells did not alter macrophage colony formation or proliferation *in vitro*. Monocyte numbers, as well as inflammatory macrophages recruited to the peritoneal cavity, were reduced in transgenic mice that specifically over-expressed *Slfn4*, while macrophage numbers and hematopoietic activity were increased in the livers and spleens.

**Conclusions:**

*Slfn4* mRNA levels were up-regulated during macrophage activation but down-regulated during differentiation. Constitutive *Slfn4* expression in the myeloid lineage *in vivo* perturbs myelopoiesis. We hypothesise that the down-regulation of *Slfn4* gene expression during macrophage differentiation is a necessary step in development of this lineage.

## Introduction

Hematopoietic stem cells in the bone marrow have the potential to self-renew or progress into various differentiation pathways to give rise to all types of mature blood cells, a process known as hematopoiesis [1]. Blood monocytes arise through a sequential series of developmental steps in which hematopoietic stem cells commit to common myeloid progenitors, followed by granulocyte-macrophage progenitors, monoblasts and finally pro-monocytes [2]. Macrophage proliferation and differentiation is controlled by macrophage colony-stimulating factor (CSF-1) [3]. Interferons (IFNs) also play an active role in this process as demonstrated by the phenotype of IFN-β^−/−^ mice, which have a reduction in circulating macrophages and granulocytes because of defective maturation of primitive bone marrow hematopoiesis [4]. This study examines the possible role of an IFN-regulated gene family in monocytopoiesis.

The *Schlafen* (*Slfn*) family [5], [6] has been identified in every mammalian genome, and in the genome of amphibians and the chondrichthyan *Callorhinchus milii* (“elephant fish”) [7]. Ten murine (m-) and six human (h-) *Slfn* members have been identified, of which only two members (h*SLFN5*/m*Slfn5*; h*SLFN14*/m*Slfn14*) are one-to-one orthologs, consistent with rapid evolution of this family across mammalian species. h*SLFN12* and h*SLFN12like* have four orthologs in mice (m*Slfn1*, m*Slfn2*, m*Slfn3*, and m*Slfn4*), whilst h*SLFN11* and h*SLFN13* have three (m*Slfn8*, m*Slfn9*, and m*Slfn10*) [7]. All Slfn proteins contain a single N-terminal divergent AAA (ATP-ases associated with various cellular activities) domain, which is presumed to be involved in GTP/ATP binding [5], [6]. The longer isoforms (Slfn5, Slfn8, Slfn9, Slfn10, and predicted Slfn14), which represent a subgroup of the family, also contain weak but significant homology to several motifs found in members of DNA/RNA helicase superfamily I [6], suggesting involvement in DNA/RNA metabolism and/or pathogen recognition.

The *Slfn* genes are predominantly expressed in cells of the immune system and are differentially regulated during developmental processes. During T-cell development, *Slfn1* and *Slfn2* mRNA expression increased and *Slfn4* mRNA expression decreased [5], [8], whilst mRNA levels of *Slfn3*, *Slfn5*, *Slfn8*, *Slfn9* and *Slfn10* were unaltered [6]. More recently, Condamine *et al.* reported that *Slfn3* expression was elevated in CD4^+^ CD25^+^ T regulatory cells as compared to CD4^+^ CD25^−^ T cells [9]. The *Slfn* genes were also regulated during IL-6 or LIF-induced differentiation of M1 monocytic leukaemia cells; the mRNA levels of all *Slfn* genes, except *Slfn3* and *Slfn9* were up-regulated [6]. Conversely, *Slfn2* expression was down-regulated during the differentiation of FDCP-1 cells into erythroid, monocytic, neutrophilic, megakaryocytic, basophilic, and eosinophilic cells [10].

In addition to regulation during cell development, *Slfn* gene expression was also modulated during bacterial infections [6], [11], [12], which suggests a role for this gene family in host defence. The first non-redundant function of a *Slfn* family member was described recently by Berger *et al.*, who reported a loss of function *Slfn2* mutation (dubbed *elektra*) that caused reduced numbers of T cells and monocytes, and increased susceptibility to bacterial and viral infections comparable to that seen with a mutation in *Myd88*, which encodes an adaptor protein in the Toll-like Receptor (TLR) pathway [13]. The monocyte defect was characterised by a disproportionate loss of Ly6C^+^ inflammatory monocytes [13], implying that *Slfn2* is required for the maturation and/or survival of this monocyte sub-set. Katsoulidis *et al.* [14] have also implicated *Slfn2* in hematopoietic development; they reported that *Slfn2* knockdown promoted mouse hemopoietic colony formation and impaired the growth suppressive actions of IFN-α.

Gain-of-function studies also suggest a role for *Slfn* family members in cell proliferation, development and differentiation. For example, ectopic expression of *Slfn1* or *Slfn8* in all thymocyte lineages *in vivo* perturbed thymocyte development [5], [6], and *Slfn1* has also been shown to cause cell cycle arrest *in vitro* by repressing mitogen-inducible cyclin D1 expression [5], [15]. Despite these recent studies, very little is known about the specific molecular functions of the *Slfn* family in macrophages. We therefore set out to profile the regulated expression patterns of *Slfn* family members in this lineage. In doing so, we showed that *Slfn4* was strikingly regulated during macrophage activation and differentiation. Utilising a novel binary system for tissue-specific gene expression, we also show that ectopic expression of *Slfn4* in cells of the myeloid lineage *in vivo* does not alter cell proliferation, but nevertheless disrupts normal myelopoiesis.

## Results

### Up-regulation of *Slfn* mRNA expression in activated bone marrow-derived macrophages (BMM)

**Figure 1:**
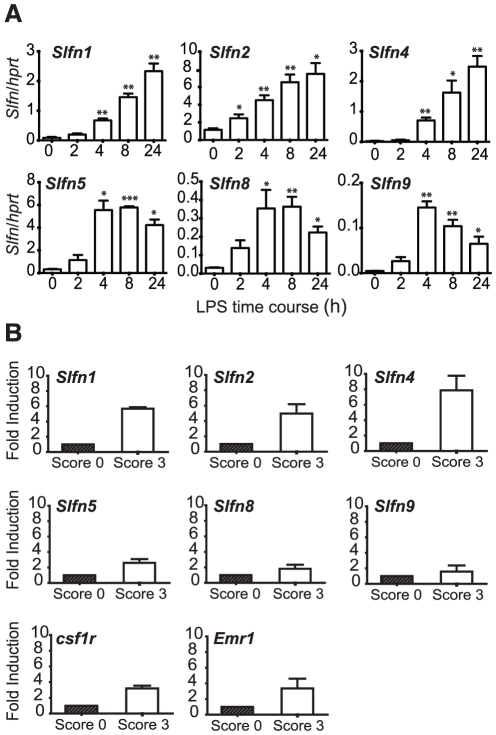
Induction of *Slfn* expression by LPS in BMM, and in the CIA mouse model. (A) BMM were stimulated with LPS over a time course (0h no treatment control, 2h, 4h, 8h, 24h), RNA was extracted and reverse transcribed, and the expression of *Slfn1*, *Slfn2*, *Slfn4*, *Slfn5*, *Slfn8*, and *Slfn9* was determined using quantitative real-time PCR. Data (mean + SEM) are combined from 3 independent experiments. * *P*<0.05 compared to control; ** *P*<0.01 compared to control; *** *P*<0.001 compared to control. (B) RNA from whole joints (disease affected, score 3; or unaffected, score 0) from mice with CIA was used to synthesise cDNA. mRNA expression of *Slfn1*, *Slfn2*, *Slfn4*, *Slfn5*, *Slfn8*, *Slfn9*, *csf1r*, and *Emr1* was determined using real-time PCR. The data, displayed as fold induction relative to control (score 0), are combined from two independent experiments (mean + range).

We began our studies on *Slfn* expression and function in macrophages by assessing the mRNA expression of those family members that could easily be discriminated by quantitative real-time PCR, in primary mouse BMM stimulated with the TLR4 agonist, LPS. Upon addition of LPS, *Slfn1*, *Slfn2*, and *Slfn4* mRNA levels were increased at all time points examined, with maximal induction after 24 hours LPS treatment. Peak induction for *Slfn5*, *Slfn8*, and *Slfn9* was more rapid, with maximal up-regulation being apparent at 4h post-LPS treatment (Figure 1A). These data indicate distinct kinetics in regulation between members that do (*Slfn5*, *Slfn8*, and *Slfn9*) or do not (*Slfn1*, *Slfn2*, and *Slfn4*) contain the helicase homology region. The most strikingly regulated gene in terms of fold response was *Slfn4* (∼200 fold). The induction of multiple *Slfn* genes in primary macrophages detected by qRT-PCR confirms comparative microarray data that we have placed in the public domain [16] (see also biogps.gnf.org).

To confirm the relevance of the induction of *Slfn* genes in macrophages to inflammatory processes, we examined gene expression in a well-described macrophage-mediated pathology, the mouse collagen-induced arthritis (CIA) model of rheumatoid arthritis (Figure 1B). The expression of the macrophage genes *Emr1* (*F4/80*) and *csf1r* was increased in CIA-affected joints, consistent with the association between macrophage infiltration and joint destruction and disease progression [17]. In this model, mRNA levels of *Slfn* family members that do not contain a helicase homology domain (*Slfn1*, *Slfn2*, *Slfn4*) were all elevated, with *Slfn4* mRNA expression increased ∼8 fold compared to joints that were not disease-affected (Figure 1B). Hence, *Slfn4* mRNA levels were strongly up-regulated in both *in vitro* (Figure 1A) and *in vivo* (Figure 1B) inflammation models.

### Promoter analysis of the murine *Slfn4* locus

**Figure 2:**
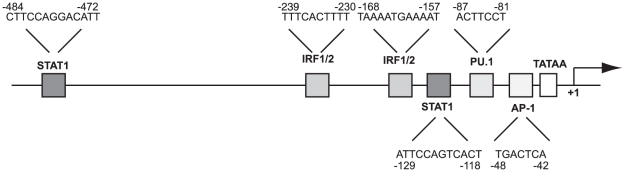
Putative regulatory elements within the *Slfn4* promoter. The *Slfn4* proximal promoter is a TATA-containing promoter with putative binding sites for STAT1, IRF family members, PU.1 and AP1. The TSS, designated +1, is marked with an arrow.

Genome scale analysis of promoter usage by cap analysis of gene expression (CAGE) has mapped the large majority of mouse transcription start sites (TSS) [18]. Amongst the many cell types analysed were BMM treated with LPS. Figure 2 shows a schematic representation of the *Slfn4* major TSS and proximal promoter region as defined by CAGE analysis (the primary FANTOM 3 CAGE data can be accessed through fantom3.gsc.riken.jp). The TSS starts on the expected pyrimidine/purine initiator [18], and lies downstream of a TATA box. Scanning of the promoter region with JASPAR (jaspar.cgb.ki.se) reveals sites that conform to the position weight matrix for AP1 and PU.1 and two copies of IFN-responsive elements, STAT1 and IRF1. These sequences suggest that *Slfn4* is a likely target of IFN-dependent signaling pathways downstream of TLRs [19].

### TLR agonists up-regulate *Slfn4* mRNA via the autocrine action of type I IFN

**Figure 3:**
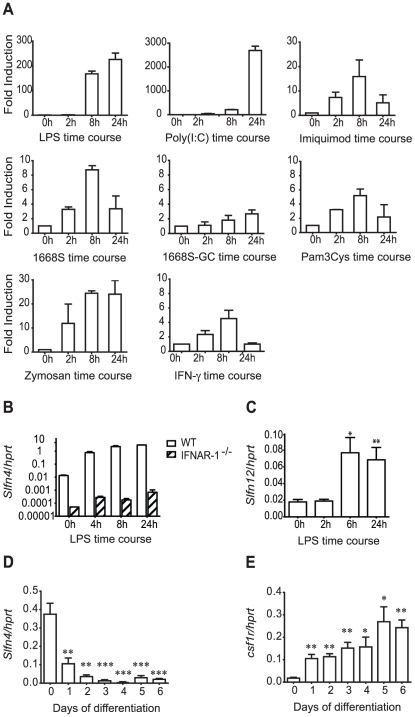
*Slfn4* mRNA is induced via autocrine type I IFN and is down-regulated during macrophage differentiation. (A) BMM were stimulated with LPS (10 ng/ml), dsRNA Poly(I∶C) (30 µg/ml), Imiquimod (15 µg/ml), Zymosan (150 µg/ml), synthetic phosphorothioate CpG oligonucleotide 1668S (0.3 µM) and non-stimulatory control oligonucleotide 1668S-GC (0.3 µM), Pam3Cys (15 ng/ml), or IFN-γ (500 pg/ml) over a 24h time course. The data, displayed as fold induction relative to control (0h), are combined from two independent experiments (mean + range). (B) BMM from IFNAR-1^−/−^ and wild type mice were stimulated with LPS over a time course. The data are combined from two independent experiments (mean + range). C) HMDM were stimulated with LPS over a time course (0h no treatment control, 2h, 6h, 24h). The data are combined from eight independent donors (mean + SEM). (D, E) Bone marrow progenitors were differentiated into BMM in the presence of CSF-1 over a 6-day time course. Data are combined from three independent experiments (mean + SEM). *Slfn4* (A–D) and *csf1r* (E) mRNA expression was determined using quantitative real-time PCR. * *P*<0.05 compared to 0h or day 0 control; ** *P*<0.01 compared to 0h or day 0 control; *** *P*<0.001 compared to 0h or day 0 control.

In macrophages, TLR4 and TLR3 ligands utilize MyD88-independent signalling to induce the type I IFN, IFN-β, which then acts as an autocrine factor. IFN-β is not produced in this cell type in response to TLR ligands that exclusively use the MyD88 adaptor protein. To test the prediction from the promoter analysis that *Slfn4* is a target of autocrine type I IFN, we examined *Slfn4* mRNA regulation in BMM stimulated with a variety of TLR agonists. We also examined the effects of IFN-γ, a pro-inflammatory cytokine that synergizes with TLR agonists in macrophage activation [20], since this cytokine has been reported to regulate mRNA stability via MyD88 [21]. As shown in Figure 3A, each of the different TLR agonists up-regulated the expression of *Slfn4* mRNA, but the magnitude of the response differed greatly. Agonists of TLR4 (LPS) and TLR3 (poly(I∶C)) caused the highest induction of *Slfn4* mRNA expression (>200 fold and >2000 fold respectively) compared to the up-regulation by other TLR agonists or IFN-γ (5–25 fold). TLR3- and TLR4-mediated activation of TRIF in a MyD88-independent fashion promotes transcription of IFN-β, which in turn act in an autocrine fashion via the type 1 IFN receptor (IFNAR) to induce the expression of downstream target genes [19]. To confirm the intermediate role of IFN, we examined IFNAR-1 null mice. Interestingly, the mutant mice expressed lower basal levels of *Slfn4* in BMM. Whereas LPS strongly up-regulated *Slfn4* mRNA expression in BMM from wild-type mice (∼300 fold), this response was dramatically reduced in IFNAR-1^−/−^ BMM (∼20 fold, Figure 3B). These findings confirm that *Slfn4* is a type I IFN target gene, but indicate also that it contains some IFN-independent responsive elements. It is possible that the perfect AP1 site highlighted in Figure 2 contributes to type I IFN-independent responses.

The *Slfn* gene families of mouse and human, like other IFN-responsive families such as the HIN200s [22], are very different in terms of copy number and genomic organization [7]. We therefore asked whether either of the human genes most closely related at a sequence level to *Slfn4* (h*SLFN12* and h*SLFN12like*) were regulated in human macrophages. h*SLFN12like* mRNA was barely detectable in primary human monocyte-derived macrophages (HMDM) and was not consistently regulated by LPS in HMDM from different donors (data not shown). In contrast, h*SLFN12* mRNA expression was up-regulated by LPS in HMDM from multiple independent donors, with maximal induction at the latest time point examined (∼4–5 fold, Figure 3C). This finding suggests that h*SLFN12* could be a functional ortholog of m*Slfn4*.

### *Slfn4* mRNA is down-regulated during macrophage differentiation

Type I IFN inhibits CSF-1-mediated proliferation of macrophages *in vitro* [23], but it is essential for normal myelopoiesis *in vivo* [4]. In the light of the observation that the *Slfn2* gene mutation alters monocyte maturation [13], as well as the fact that *Slfn4* is a type I IFN-dependent gene in BMM, we examined *Slfn4* mRNA levels during CSF-1-mediated differentiation of bone marrow progenitors into BMM. *Slfn4* mRNA was highly expressed in unstimulated bone marrow cells (day 0), but rapidly declined in response to CSF-1 (Figure 3D). As expected, *csf1r* mRNA levels showed an opposing profile, with mRNA levels increasing during differentiation (Figure 3E). Consistent with our hypothesis that h*SLFN12* may be a functional ortholog of m*Slfn4*, expression profiling data on biogps.gnf.org revealed that h*SLFN12* mRNA is more highly expressed in CD33-positive myeloid progenitors than in CD14^+^ monocytes.

### Ectopic expression of *Slfn4* in cells of the mononuclear phagocyte system *in vivo* dysregulates myelopoiesis

**Figure 4:**
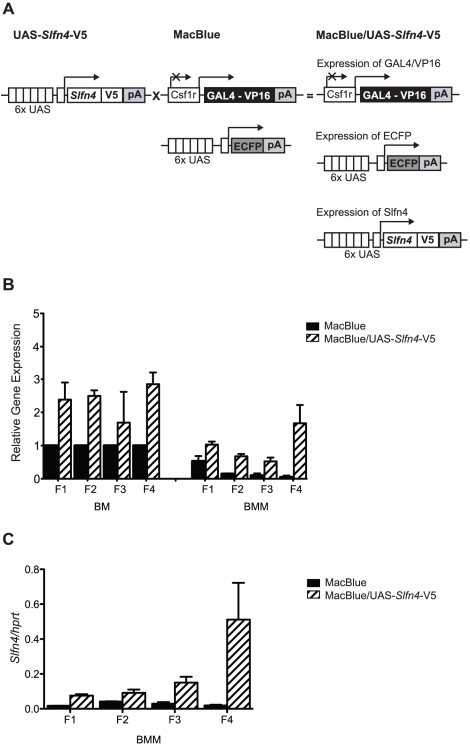
Macrophage-specific expression of the UAS-*Slfn4*-V5 transgene. (A) Elements of the UAS-*Slfn4*-V5 transgene include six UAS, a kozak sequence, and the open reading frame of *Slfn4* followed by a V5-tag. Upon crossing of the UAS-*Slfn4*-V5 mouse with the MacBlue mouse, the offspring (MacBlue/UAS-*Slfn4*-V5 mice) contain the GAL4-expressing module, the GAL4-reporting module, and the UAS-*Slfn4*-V5 transgene. GAL4/VP16 protein binding to both the UAS induces the expression of ECFP and *Slfn4*-V5 specifically in cells of the myeloid lineage. (B and C) RNA from bone marrow (BM) or BMM from the offspring of four UAS-*Slfn4*-V5 founder lines (F1–F4) was extracted and cDNA was prepared. *Slfn4* mRNA levels relative to *hprt* were determined by quantitative real-time PCR. Data are combined from at least two independent experiments (mean + range) and are presented as expression relative to BM controls to enable normalization across different the transgenic lines (B), or as expression relative to *hprt* (no normalization across the transgenic lines) (C).

The *Slfn* gene family is expanded in mice [7], and non-helicase-containing *Slfn* family members showed a similar pattern of regulation in macrophages (Figure 1). We therefore hypothesized that related family members may render *Slfn4* functionally redundant and that genetic deletion of this gene may not necessarily reveal a phenotype. Therefore, to determine the significance of down-modulation of *Slfn4* during macrophage differentiation, and/or its induction by LPS and IFN, we expressed the gene constitutively in myeloid cells. To do so, we employed a novel binary system, the *Csf1r*-GAL4VP16/UAS-ECFP mice (MacBlue mice) [24]. In MacBlue mice, the myeloid-restricted *csf1r* promoter drives expression of the GAL4-VP16 transcriptional activator, which in turn binds to the UAS repeats upstream of ECFP to drive expression of ECFP in myeloid cells (Figure 4A). MacBlue transgenic mice can also be utilised for macrophage-specific over-expression of UAS-containing GAL4-dependent transgenes [24]. The *csf1r* promoter used in the MacBlue mice has an internal deletion that abolishes expression in placental trophoblasts and osteoclasts, and reduces expression in mature macrophages and granulocytes [25]. We generated four independent UAS-*Slfn4*-V5 founder lines to mate with the MacBlue mice to generate double transgenics (MacBlue/UAS-*Slfn4*-V5 transgenic mouse, Figure 4A). BMM from double-transgenics from the four UAS-*Slfn4*-V5 founder lines expressed 2, 4, 5 and 20 fold higher levels of *Slfn4* mRNA than BMM from MacBlue littermate controls (Figure 4B), effectively providing an allelic series. The levels of *Slfn4* mRNA were already high in bone marrow cells, and were only moderately elevated in bone marrow from MacBlue/UAS-*Slfn4*-V5 mice as compared to littermate controls (UAS-*Slfn4*-V5 and MacBlue mice) (Figure 4B). Despite the transgene expression, *Slfn4* mRNA expression was still down-regulated in BMM as compared to bone marrow cells in all of the lines (Figure 4B), as we observed previously (Figure 3D). Thus, the levels of *Slfn4* mRNA over-expression remain within a physiological range in the transgenic animals. In effect, the transgenes generated the desired outcome of maintaining *Slfn4* mRNA in macrophages at similar levels to those present in their progenitors (Figure 4B). It should be noted that these data are presented as expression relative to the bone marrow cells from littermate controls for each line, so that the general expression patterns across the different transgenic lines can be compared. The actual levels of *Slfn4* mRNA expression did vary between the different lines. Figure 4C shows that *Slfn4* mRNA levels in BMM from founder F4 were particularly elevated as compared to the other three lines.

### Decreased monocyte and elicited macrophage numbers in *Slfn4* over-expressing mice

To determine whether the sustained expression of *Slfn4* has any impact on myelopoiesis, peripheral blood leukocytes, thioglycollate-elicited peritoneal macrophages (TEPM), and bone marrow were isolated and immunostained for the surface markers CSF1R and F4/80 (monocyte/macrophage markers), Ly-6G (granulocyte marker) or B220/CD45R (B cell marker) (Table 1). There was no significant difference in the percentage of CSF1R, F4/80, B220/CD45R or Ly-6G expressing cells in bone marrow from MacBlue/UAS-*Slfn4*-V5 mice compared to littermate controls. However, the percentage of CSF1R, F4/80, and ECFP expressing cells (monocytes) was approximately 50% reduced in peripheral blood from MacBlue/UAS-*Slfn4*-V5 mice, whilst the number of Ly-6G positive cells (granulocytes) was unaffected. Reduced monocyte numbers were observed in independent *Slfn4* transgenic lines, indicating that the phenotype was unlikely to arise from insertional effects of the transgene. For the transgenic line with the highest level of *Slfn4* expression (Figure 4C), we also monitored recruitment of inflammatory macrophages to the peritoneal cavity. A statistically significant reduction in CSF1R, F4/80, and ECFP expressing cells was observed in TEPM from MacBlue/UAS-*Slfn4*-V5 mice, as compared to littermate controls (Table 1). Furthermore, thioglycollate-elicited peritoneal exudates from *Slfn4* transgenic mice contained 2–3 fold less cells than littermate controls, and most of these cells were granulocytic in appearance (data not shown). These findings suggest that ectopic expression of *Slfn4* in *csf1r*-positive cells dysregulates myelopoiesis, and consequently, the recruitment of inflammatory macrophages.

**Table 1:** Decreased monocyte and elicited macrophage numbers in Slfn4 over-expressing mice.

	UAS-*Slfn4*-V5	MacBlue	MacBlue/UAS-*Slfn4*-V5
BM (%)			
CSF1R	10.1±2.5	10.9±2.8	11.3±3.5
F4/80	20.5±4.9	20.2±4.1	20.6±4.8
Ly-6G	40.2±9.7	36.5±10.4	54.0±4.8
B220 (CD45R)	23.7±2.4	21.1±0.8	17.9±0.6
ECFP	-	12.0±1.7	11.1±0.4
PBL (%)			
CSF1R	9.5±0.1	8.2±1.8	2.8±1.5*
F4/80	9.7±0.2	10.1±1.7	4.3±1.0*
Ly-6G	36.8±0.6	35.2±4.7	33.8±0.9
ECFP	-	11.7±2.2	5.1±0.9*
TEPM (%)			
CSF1R	56.3±2.9	49.8±2.1	22.8±3.5 **
F4/80	59.9±4.4	43.9±2.8	20.9±4.3 *
ECFP	-	44.2±0.9	28.8±2.8 **

BM, bone marrow; PBL, peripheral blood leukocytes; TEPM, thioglycollate-elicited peritoneal macrophages. FACS data are combined from at least three independent experiments. Data represent mean ± SEM. For BM and PBL, data were collected from two independent transgenic lines and littermate controls. For TEPM, data were generated from the transgenic line with the highest transgene expression (F4) and littermate controls.

*P<0.05;

**P<0.01.

### *Slfn4* over-expression did not alter bone marrow proliferation or cell viability

**Figure 5:**
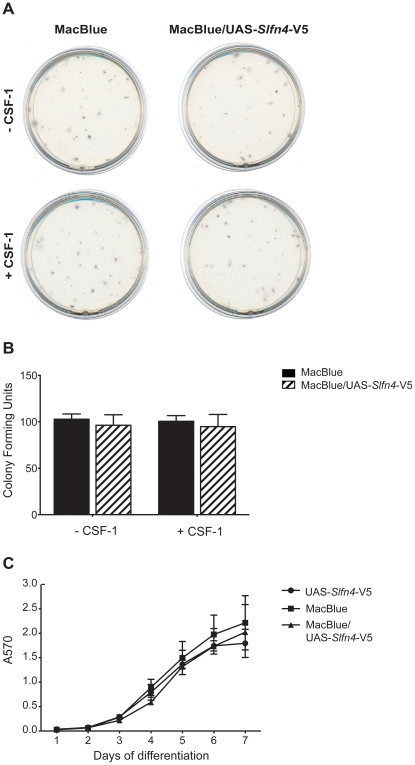
Slfn4 over-expression did not alter bone marrow proliferation or cell viability. Bone marrow cells from MacBlue/UAS-*Slfn4*-V5 mice and littermate controls were cultured in semisolid methylcellulose medium in the presence (+) or absence (−) of CSF-1. The size (A) and frequency (B) of colonies were examined on day 14. Data are combined from six independent experiments and are displayed as mean + SEM. (C) Bone marrow cells from MacBlue/UAS-*Slfn4*-V5 mice and littermate controls were cultured in the presence of CSF-1 over a 7-day time course and cell survival was measured on each day by MTT assay. Data are combined from three independent experiments and are displayed as mean ± SEM.

A steady-state reduction in blood monocytes could result from a decrease in monocyte production in the marrow, or a reduction in circulating half-life. As noted in the introduction, other *Slfn* family members when over-expressed inhibit growth. To determine whether there was any impact of *Slfn4* on either the frequency or proliferation of hematopoietic progenitor cells, CFU assays were performed with bone marrow from MacBlue/UAS-*Slfn4*-V5 mice and littermate controls. Bone marrow progenitor cells were cultured *in vitro* in semisolid methylcellulose medium to examine CFU-GM. Bone marrow progenitor cells were also cultured in the presence of CSF-1 to assess the effect of *Slfn4* over-expression on high proliferative potential colony-forming cells, which produce very large colonies (diameter >0.5 mm) when cultured with IL-3 plus CSF-1 due to an increase in CSF-1R expression [26], [27]. There was no significant difference in size (Figure 5A) or frequency (Figure 5B) of CFU-GM and high proliferative potential colony-forming cells from MacBlue/UAS-*Slfn4*-V5 bone marrow compared to MacBlue bone marrow. This finding is not entirely surprising given that the transgene does not greatly increase the already high expression of *Slfn4* in bone marrow cells. We therefore examined macrophage proliferation in liquid culture. There was no impact of the MacBlue/UAS-*Slfn4*-V5 transgene on the time course, or the ultimate yield of viable cells, in liquid cultures of bone marrow cells in CSF-1 (Figure 5C). These data suggest that some factor required for the *in vivo Slfn4* phenotype is not present in the *in vitro* conditions used here.

### *Slfn4* over-expressing animals display splenomegaly

**Figure 6:**
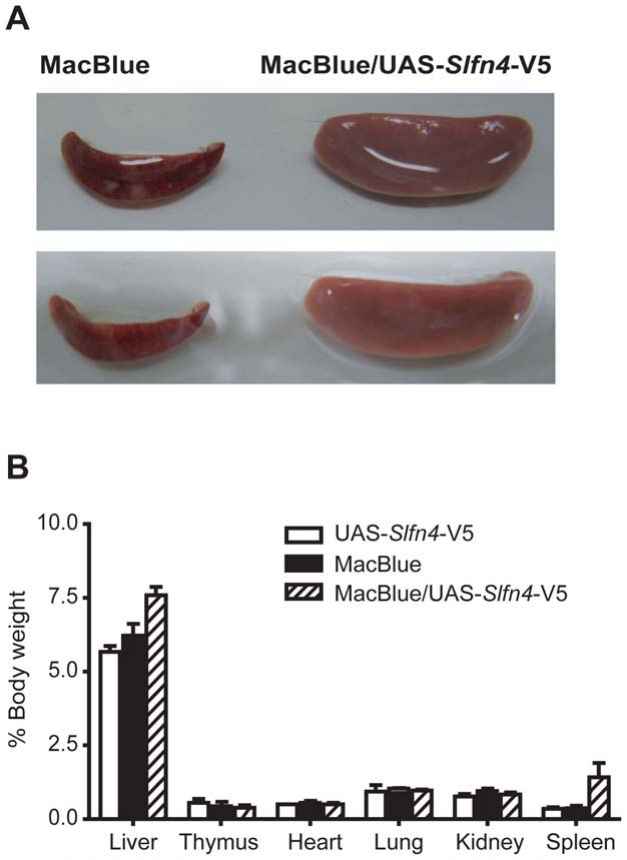
Slfn4 over-expression caused splenomegaly. (A) Macroscopic appearance of spleens from *Slfn4* over-expressing mice (right) and from MacBlue littermate controls (left). (B) Organ weights are expressed as percentage of body weight. Data are combined from four independent experiments and are displayed as mean + SEM.

**Figure 7:**
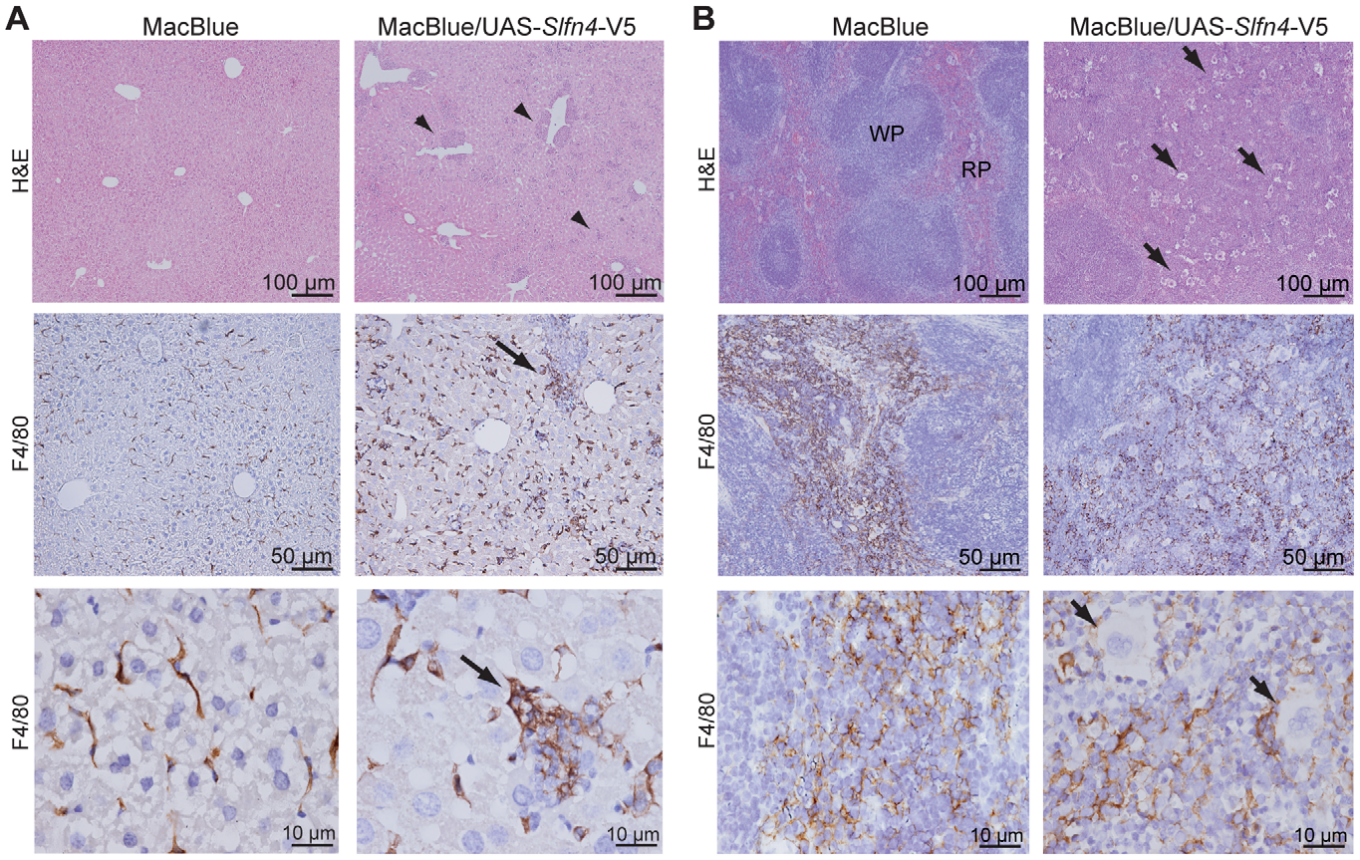
Increased neutrophils and macrophages in the livers and spleens of MacBlue/UAS-*Slfn4*-V5 mice. (A) Hematoxylin and eosin staining was performed on liver paraffin sections. Arrowheads indicate aggregates of neutrophils in the liver. Liver sections from MacBlue/UAS-*Slfn4*-V5 and MacBlue littermate controls were also stained for F4/80 (brown). Arrows indicate F4/80 expressing cells surrounding aggregates of neutrophils. (B) Hematoxylin and eosin staining was also performed on spleen paraffin sections. Arrows indicate clusters of megakaryocytes. Spleen sections from MacBlue/UAS-*Slfn4*-V5 and MacBlue littermate controls were also stained for F4/80 (brown). Sections were examined using an Olympus BX-51 microscope with a DP-70 digital camera and DP controller imaging software (Olympus). RP, red pulp; WP, white pulp.

MacBlue/UAS-*Slfn4*-V5 mice with the highest level of transgene expression (20 fold over control, F4; Figure 4C) displayed splenomegaly; spleens were diffusively and symmetrically enlarged (Figure 6A), and there was a 3 to 4 fold increase in splenic weight relative to the total body weight compared to littermate controls (Figure 6B). There was also a trend towards an increase in liver weight, but no difference in weight or macroscopic appearance of other tissues examined including thymus, heart, lung, and kidney (Figure 6B). Histological examination of organs from MacBlue/UAS-*Slfn4*-V5 mice revealed an increase in neutrophils in the liver (Figure 7A, arrowheads), as well as an increase in neutrophils and megakaryocytes in the spleens (Figure 7B, arrows). The clear definition of red and white pulp in the spleen was also lost in these mice (Figure 7B), whilst the cellularity or morphology of other tissues from *Slfn4* over-expressing animals was not altered (data not shown). Staining of liver and spleen sections with the macrophage marker F4/80 also revealed an increase of macrophages, particularly in liver, which often surrounded aggregates of neutrophils (Figure 7A, arrow).

**Figure 8:**
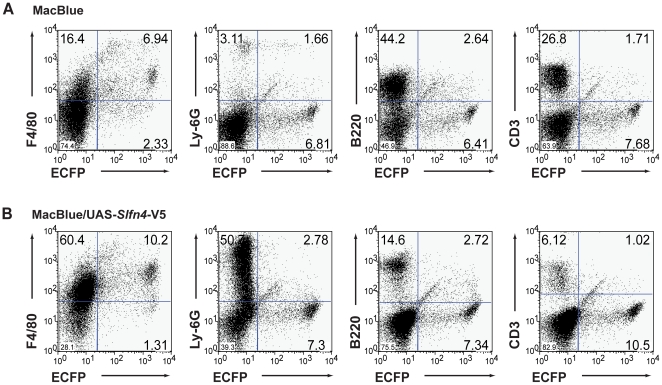
Increased macrophages and granulocytes in spleen from MacBlue/UAS-*Slfn4*-V5 mice. Splenocytes from MacBlue littermate controls (A) and MacBlue/UAS-*Slfn4*-V5 mice (B) were stained for the cell surface markers F4/80 (macrophage), Ly-6G (granulocyte), B220 (CD45R, B cell), and CD3ε (T cell). The samples were analysed by FACS and quadrants were set based upon isotype control profiles. Data are representative of two independent experiments.

To dissect the phenotype of the myeloid infiltrate further, we disaggregated the spleen and stained for myeloid markers. The MacBlue marker (ECFP) itself provides a marker of myeloid maturation because of the promoter deletion, which restricts expression to mature macrophages [24], [25]. There was an ∼3 fold increase in the percentage of cells expressing the monocyte/macrophage-specific F4/80 marker, and an ∼10 fold increase in the percentage of cells expressing the granulocyte-specific Ly-6G marker in the enlarged spleens from MacBlue/UAS-*Slfn4-*V5 mice compared to littermate control mice (Figure 8). Correspondingly, the percentage of B220/CD45R-expressing B cells and CD3ε-expressing T cells were both decreased (∼3–4 fold) (Figure 8). Interestingly, the percentages of splenic macrophages expressing both F4/80 and ECFP, and granulocytes expressing both Ly6G and ECFP, were increased only marginally. One possibility is that *Slfn4*-positive progenitors are selected against, with concomitant expansion of *Slfn4*-negative progenitors in the spleen. In support of this idea, we did not detect significant numbers of CSF-1R^+^ ECFP^−^ cells in the spleen (data not shown), suggesting that the expanded myeloid ECFP^−^ cells in the spleen did not express *Slfn4*. In summary, a threshold level of ectopic *Slfn4* expression appears to generate a form of myelodysplasia, because the phenotype was not observed in lines expressing lower levels of *Slfn4*. Given that the splenomegaly phenotype was apparent in only one line however, we cannot completely exclude the possibility that insertional effects generate this phenotype.

## Discussion

The limited studies thus far performed on the *Slfn* genes suggest a role in cell growth and differentiation. Indeed, very recent studies have implicated *Slfn2* and *Slfn3* in the differentiation of monocytes [13] and the intestinal epithelium [28], [29] respectively. We showed that *Slfn4* mRNA expression was substantially reduced during CSF-1-mediated macrophage differentiation (Figure 3D), and that numbers of monocytes and recruited inflammatory macrophages were reduced in mice constitutively expressing *Slfn4* in the myeloid lineage (Table 1). The most obvious interpretation of these data is that *Slfn4* is a negative regulator of monocytopoiesis. We also observed increased numbers of macrophages in the livers and spleens of *Slfn4* transgenic mice (Figure 7, 8). Given this observation, an alternative explanation is that enforced *Slfn4* expression does not affect monocyte production, but causes dysregulated monocyte trafficking, which results in increased macrophage numbers in these organs. We cannot discount this possibility, but several lines of evidence suggest that this phenotype is more likely to relate to disrupted myelopoiesis. Firstly, we observed increased numbers of granulocytes and megakaryocytes, not just macrophages, in the spleens of *Slfn4* transgenic mice (Figure 7, 8). Secondly, the normal splenic architecture was disrupted (Figure 7). These are both features of extramedullary haematopoiesis, typically associated with a defect in normal haematopoiesis. Of note, the increased granulocyte and macrophage populations in the spleens of *Slfn4* transgenic mice were predominantly negative for the UAS-dependent CFP transgene (Figure 8). Therefore, if there is defective monocyte trafficking, it is unlikely to result from cell-autonomous effects of *Slfn4*-expressing monocytes, since this transgene is also UAS-dependent. A more likely explanation is that, in the spleens of *Slfn4* transgenic mice, there is a preferential expansion of a sub-set of myeloid progenitors that do not ectopically express *Slfn4*. This is also supported by our observation that it was essentially only the ECFP^+^ cells that were CSF-1R^+^ in the spleens of these mice (data not shown), again suggesting that the expanded myeloid population do not express *Slfn4*. Together with our data on gene regulation during macrophage differentiation (Figure 3D), the phenotypes of the *Slfn4* transgenic mice (Table 1, Figure 7, Figure 8) lead us to hypothesize that down-regulation of *Slfn4* expression is a necessary step required for normal differentiation along the monocyte/macrophage lineage. In this scenario, enforced *Slfn4* expression in the myeloid lineage would disrupt monocytopoiesis. It should be noted that only the line with the greatest level of *Slfn4* expression (Figure 4C) displayed the splenomegaly phenotype. We therefore cannot completely exclude that this particular phenotype arises from an insertional effect of the transgene, but this would seem unlikely because the monocytopoiesis defect was apparent in multiple independent lines, and because various *Slfn* family members have been shown to regulate immune cell development and/or survival. A more likely explanation is that a threshold level of *Slfn4* expression is required to reveal the more severe splenomegaly phenotype.

The molecular mechanisms by which *Slfn4* regulates monocyte/macrophage development and/or function are unclear. When expressed in HEK293T cells, helicase-containing Slfns were present in the nucleus, whilst Slfn2 and Slfn4 (non-helicase-containing Slfns) were exclusively cytoplasmic [30]. Similarly, we found that Slfn4 was localized to the cytoplasm when ectopically expressed in RAW264 mouse macrophages (WvZ, unpublished data). Slfn family members all contain an AAA domain, which is typically associated with protein oligomerization. One possibility is that Slfn4 oligomerizes with Slfn2 since they are present in the same cellular compartment [30]. In contrast to the down-regulation of *Slfn4* mRNA expression during macrophage differentiation that we observed, expression of *Slfn2* was up-regulated during myeloid differentiation [30]. Consistent with this, *Slfn2* mRNA is expressed at elevated levels in mature macrophages, as well as T cells (biogps.gnf.org). As opposed to the negative regulatory role of Slfn4 in myelopoiesis that we propose, functional Slfn2 was actually required for monocyte and T cell maturation [13]. Hence, one possibility is that Slfn4 interacts with Slfn2, and inhibits its function. In this setting, down-regulation of *Slfn4* gene expression would permit terminal macrophage differentiation by removing the block on Slfn2 action. Whatever the case, our understanding of the mechanisms by which Slfn4 and other members of the family regulate monocyte/macrophage function is hindered by the current lack of knowledge of Slfn functions at the molecular level, and by the fact that AAA domain-containing proteins have diverse roles in a multitude of biological processes.

Phylogenetic analysis of the *Slfn* genes indicated that the *Slfn* gene region, located in an otherwise conserved human-mouse interval, is evolving rapidly, possibly in response to selective pressure related to their function in the immune response [7]. It therefore remains an open question as to how conserved the biology of *Slfn* genes is between humans and mice. Nonetheless, the findings that LPS also up-regulated the mRNA expression of h*SLFN12* (Figure 3C), but not h*SLFN12like* (data not shown) in HMDM, and that h*SLFN12* mRNA was elevated in CD33-positive myeloid progenitors compared to monocytes (biogps.gnf.org), indicates that m*Slfn4* and h*SLFN12* show similar regulation. We therefore speculate that these 2 genes may also have conserved functions in mice and humans respectively; future functional studies on hSLFN12 may reveal roles in human macrophage differentiation. For example, it is possible that the myelodysplastic syndrome associated with copy number gains at Chr 17q12 [31] relates to elevated h*SLFN12* expression and function, since this gene is located in this region.

In addition to regulating monocyte/macrophage development, *Slfn4* is likely to have some role in regulating macrophage activation, given the effect of TLR3 and TLR4 agonists on *Slfn4* mRNA expression. TLR-inducible expression involves the autocrine, type I IFN-dependent pathway, but there was some residual induction in IFNAR-1^−/−^ macrophages (Figure 3B), and by agonists that act in a strictly MyD88-dependent manner (Figure 3A). The role of type I IFN in the induction of *Slfn4* is consistent with the presence of appropriate response elements in the promoter (Figure 2). A major function of IFNs is to protect cells against viral infection by inducing a suite of genes involved in host defence [32]. As a type I IFN-inducible gene, *Slfn4* may play some role in responses to viral infections. Consistent with this, *Slfn4* was induced in macrophages in response to adenovirus infection (AI, unpublished data), and microarray data published by others reported up-regulation of *Slfn4* mRNA in BMM upon infection with Sendai virus [33] and in the lungs of influenza A/PR/8/34 virus infected mice [34]. Another link between Slfns and responses to viral infections is suggested by the similarity between these genes and the right inverted terminal repeat of several *orthopoxviruses* [5], [35]. Somewhat surprisingly, *Slfnlike* protein 176 from Camelpox strain CMS apparently reduces virulence, because mice infected with recombinant vaccinia and variola viruses expressing *v-Slfn* recovered sooner than mice infected with non-recombinant virus [35]. What functional role Slfn4 has in anti-viral responses remains to be determined. Unfortunately, we were not able to assess the impact of the *Slfn4* over-expression on host defence because of the myelodysplastic phenotype of *Slfn4* transgenic mice. This would render pathogen challenge experiments uninterpretable. Furthermore, given that our *in vitro* experiments assessing macrophage differentiation did not reveal a phenotype despite the fact that *Slfn4* regulated monocytopoiesis *in vivo*, a yet to be identified *in vivo* factor may be required for *Slfn4* function *in vitro*. Consistent with this, expression profiling of LPS-activated *Slfn4* over-expressing macrophages versus controls did not reveal any consistent effect of constitutive *Slfn4* expression on the TLR4 response (data not shown). Consequently, the role of *Slfn4* in responses to TLR agonists and pathogen challenge in this *in vitro* setting may require the identification of such a factor.

LPS robustly induced all members of the Slfn family that were examined (Figure 1A). *Slfn1*, *Slfn2* and *Slfn4*, which all lack the C-terminal helicase-like domain, were late LPS response genes in mouse macrophages, whilst the induction of helicase-containing *Slfns* (*Slfn5*, *8* and *9*) peaked earlier and declined somewhat by 24 h (Figure 1A). Many TLR-inducible genes that are induced with delayed kinetics act as negative feedback regulators of TLR signalling by inhibiting the function of related isoforms [36]. We therefore speculate that *Slfn5*, *8* and *9* are induced by TLR agonists to perform specific helicase-dependent functions, and the subsequent induction of non-helicase-containing Slfns permits feedback regulation to switch off the response. If this is true, then the elevated expression of *Slfn1*, *Slfn2* and *Slfn4* in CIA disease tissue (Figure 1B) may represent a host attempt to dampen inflammation. Whatever the exact functions of the short *Slfn* isoforms in rheumatoid arthritis and other inflammatory diseases, our expression analysis data are consistent with microarray analysis from two other murine models of arthritis, human T-cell leukemia virus type I transgenic mice and interleukin-1 receptor antagonist knockout mice [37].

In summary, we have shown that *Slfn4* mRNA expression is dynamically regulated during macrophage activation and differentiation. Using a novel binary system for myeloid-specific transgene expression that we previously developed for CFP [24], we have now demonstrated that this system also permits *in vivo* analysis of gene function in macrophages. From the combined data that we present here, we hypothesize that down-regulation of *Slfn4* expression during differentiation permits appropriate development along the monocyte/macrophage lineage, that *Slfn4* (as well as other *Slfn* family members) are involved in macrophage responses to pathogen challenge, and that h*SLFN12* has similar functions in human macrophages.

## Materials and Methods

### Ethics Statement

Prior to undertaking the studies described, approval for all experiments using mice was obtained from the University of Queensland Animal Ethics Committee (Project numbers: IMB/494/07 and IMB/867/08). Approval for all experiments using primary human cells was obtained from the University of Queensland Medical Research Ethics Committee (Project numbers: 2007000755 and 2009001051).

### Cell Culture and Reagents

RAW264.7 macrophage-like cells, obtained from the American Type Culture Collection (ATCC, Rockville, MD, USA), were cultured in RPMI 1640 medium (Invitrogen, Carlsbad, CA) supplemented with 5% FCS (JRH Biosciences Inc, Lenexa, Ks), 20 U/ml penicillin (Invitrogen, Carlsbad, CA), 20 µg/ml streptomycin (Invitrogen, Carlsbad, CA), and 2 mM L-glutamine (Invitrogen, Carlsbad, CA) (complete medium). BMM were obtained by *ex vivo* differentiation of mouse bone marrow cells in the presence of recombinant human CSF-1 as described previously [38]. To generate TEPM, mice were administered 1 ml 10% thioglycollate broth by i.p. injection. Cellular exudates were collected 5 days later. Peripheral blood from mice was collected by cardiac puncture in a heparin-coated syringe, and red blood cells were lysed with Red blood Lysing buffer (Sigma-Aldrich, St. Louis, MO, USA). Splenocytes were obtained by digestion of spleen with Collagenase/Dispase and DNase (Roche, Mannheim, Germany), and red blood cells were lysed with Geys solution. HMDM were obtained by *in vitro* differentiation of CD14^+^ monocytes [39]. *Salmonella minnesota* Re 595 LPS (Sigma-Aldrich, St Louis, MO) was prepared as described previously [40]. Recombinant mouse IFN-γ was purchased from R&D systems (Minneapolis, MO, USA), Pam_3_CysSerLys_4_ from Merck (Darmstadt, Germany), Zymosan from *Saccharomyces cerevisiae* was from Sigma-Aldrich (St Louis, MO), and R837 (Imiquimod) was purchased from InvivoGen (San Diego, CA, USA). Phosphorothioate-modified oligodeoxynucleotides (Geneworks, Adelaide) used were CpG DNA-activating oligonucleotide 1668S: 5′-TCCATGACGTTCCTGATGCT-3′ and the corresponding control GpC oligonucleotide 1668S-GC: 5′-TCCATGAGCTTCCTGATGCT-3′.

### CIA mouse model

DBA/1 mice were immunised with chicken type II collagen as previously described [41], and each limb was allocated a clinical score for arthritis, which was based on the following criteria; 0 = normal; 1 = light swelling; 2 = extensive swelling and/or redness of the footpad; 3 = joint distortion and/or rigidity [42]. RNA from whole joints of mice with CIA score 0 and score 3 was reverse transcribed and subjected to quantitative real-time PCR.

### Real-time PCR analysis of gene expression

RNA, prepared using Qiagen mini-kits (Qiagen, Valencia, CA), was reverse transcribed to cDNA with Superscript III reverse transcriptase (Invitrogen, Carlsbad, CA, USA). cDNA levels of all genes and the internal control, hypoxanthine-guanine phosphoribosyl transferase (*hprt*), were estimated by quantitative real-time PCR using SYBR Green (Applied Biosystems, Foster City, CA), gene specific primers, and an ABI Prism 7000 sequence detection system (Applied Biosystems). Threshold cycle (Ct) values were calculated from amplification plots and gene expression was expressed relative to the control gene, *hprt*. The primers used to detect expression of the corresponding genes are described in Table 2.

**Table 2:** Real-time PCR primers used in this study.

Gene (murine)	Forward primer	Reverse primer
*Slfn1*	5′-GCCAGACCAGCACCTGCAC-3′	5′-AAGAGGTTGGAGGGGGCTCAT-3′
*Slfn2*	5′-GCTTTAATGCAGCAAGGAACAAAGA-3′	5′-TGGGCTTTGGCACTTGGAA-3′
*Slfn4*	5′-GCCCTCTGTTCAAGTCAAGTGTCC-3′	5′-CCCAGATGAAATCCTTTCCACGA-3′
*Slfn5*	5′-TGGATAGTCTGGGTAGTCACGT-3′	5′-GTCTCAGATCCCATTGGAAT-3′
*Slfn8*	5′-CACCAGAACTGGGACCTGAGCA-3′	5′-TTAAAGGAACGCGTCGCCAAGT-3′
*Slfn9*	5′-CGGGAAGGAAAGGGAGACTTACA-3′	5′-GCTGATCCACTTTCATTTTGTGAGAA-3′
*Emr1*	5′-TCTGTGGTCCCACCTTCAT-3′	5′-GATGGCCAAGGATCTGAAAA-3′
*csf1r*	5′-CCAGAGCCCCCACAGATAA-3′	5′-AGCTTGGTGTCTCCACGTTTG-3′
*hprt*	5′-GCAGTACAGCCCCAAAATGG-3′	5′-AACAAAGTCTGGCCTGTATCCAA-3′
UAS-*Slfn4*-V5	5′-GTGCATTGGAACGCGCATTCC-3′	5′-ATCCATCTTCCTCGCTTGGCAT-3′
*Csf1r*-GAL4VP16	5′-TGGGCCTTCCGTGGCTTTGTTG-3′	5′-GCGGCCGCTAGATCTCGAGCATATCTCGAC-3′
UAS-ECFP	5′-GTGCATTGGAACGCGCATTCC-3′	5′-GCTGAAGCACTGCACGCCCC-3′

Gene (human)	Forward primer	Reverse primer
*SLFN12*	5′-TGTGGAGTGCACTTTCGTTT-3′	5′-CTTGGCGTGTTGAACAAAGA-3′
*SLFN12like*	5′-AGCCCTGAAGGCAAGCAC-3′	5′-CCAAGTCTTTCATTGTTTGAGC-3′
*hprt*	5′-TCAGGCAGTATAATCCAAAGATGGT-3′	5′-AGTCTGGCTTATATCCAACACTTCG-3′

### Generation of transgenic mice

Murine *Slfn4* cDNA was cloned into the pGene vector (Invitrogen, Carlsbad, CA, USA). To generate the upstream activating sequence (UAS)-*Slfn4*-V5 transgenic mouse line, this construct was isolated from the plasmid backbone, gel purified, and injected into pronuclei of F_2_BCB (C57BL/6JxCBA) zygotes (performed by the Transgenic Animal Service of Queensland, http://tasq.uq.edu.au). Offspring from three independent injections were screened for incorporation of the transgene by PCR amplification of tail tip genomic DNA. Identified UAS-*Slfn4*-V5 founders were crossed with *Csf1r*-GAL4VP16/UAS-enhanced cyan fluorescent protein (ECFP) mice [24], and the offspring were screened for the presence of the UAS-*Slfn4*-V5 (f, 5′-GTGCATTGGAACGCGCATTCC-3′; r, 5′-ATCCATCTTCCTCGCTTGGCAT-3′), *Csf1r*-GAL4VP16 (f, 5′-TGGGCCTTCCGTGGCTTTGTTG-3′; r, 5′-GCGGCCGCTAGATCTCGAGCATATCTCGAC-3′), and UAS-ECFP (f, 5′-GTGCATTGGAACGCGCATTCC-3′; r, 5′-GCTGAAGCACTGCACGCCCC-3′) transgenes by PCR amplification. Male and female mice of a mixed background, between 6 and 9 weeks of age were included in this study. Mouse colonies were maintained under specific pathogen-free conditions at the Institute for Molecular Bioscience, University of Queensland, Australia. This study was carried out in strict accordance with the recommendations of the relevant University of Queensland Animal Ethics Committee, and all efforts were made to ameliorate suffering.

### Organ weight and Histomorphology

Tissues from animals were harvested, and their weight and body weight were recorded. Tissues were fixed in paraformaldehyde and paraffin embedded. Sections were stained with eosin or an antibody against F4/80 (Serotec, Oxford, UK) and counterstained with Mayer's haematoxylin as described previously [43].

### Flow cytometry

Cells were washed in ice-cold 0.1% BSA, 0.1% NaN_3_ in PBS and incubated with primary antibody for 30 min. Cells were then washed (0.1% BSA, 0.1% NaN_3_ in PBS), incubated with secondary antibody for 30 min, washed with PBS, and analysed using a BD LSRII flow cytometer (BD Biosciences). AFS98, a mAb directed against the murine CSF-1 receptor was obtained from John Hamilton (University of Melbourne, Melbourne Australia). Other antibodies used were directed against F4/80 (Serotec, Oxford, UK), Ly-6G (BD Pharmingen, San Diego, CA, USA), B220 (CD45R) (BD Pharmingen, San Diego, CA, USA), or CD3ε (BD Pharmingen, San Diego, CA, USA). Secondary antibodies conjugated to PE were purchased from Serotec (Oxford, UK).

### Colony-forming unit assay

CFU assays with bone marrow cells were performed using MethoCult semisolid methylcellulose medium containing a cytokine cocktail of Stem Cell Factor, IL-6, and IL-3 (StemCell technologies, Vancouver, Canada) with or without 10^4^ U/ml CSF-1 according to manufacturer's instructions. The frequency and size of the colonies were assessed on day 14 using light microscopy.

### MTT Assay

MTT assays were conducted as previously described [38]. The absorbance was read with a Powerwave XS plate reader (Bio-Tek, Winooski, VT, USA).

### Statistical analysis

When data from multiple independent experiments were combined, the mean and SEM of the numeric data was calculated. Data were analyzed by Student's t test, where appropriate. Comparisons of data sets yielding *P* values of greater than 0.05 were regarded as not statistically different.
